# Terahertz Spin Current Dynamics in Antiferromagnetic Hematite

**DOI:** 10.1002/advs.202300512

**Published:** 2023-04-21

**Authors:** Hongsong Qiu, Tom S. Seifert, Lin Huang, Yongjian Zhou, Zdeněk Kašpar, Caihong Zhang, Jingbo Wu, Kebin Fan, Qi Zhang, Di Wu, Tobias Kampfrath, Cheng Song, Biaobing Jin, Jian Chen, Peiheng Wu

**Affiliations:** ^1^ Research Institute of Superconductor Electronics (RISE) School of Electronic Science and Engineering Nanjing University Nanjing 210023 P. R. China; ^2^ Department of Physics Freie Universität Berlin 14195 Berlin Germany; ^3^ Key Laboratory of Advanced Materials (MOE) School of Materials Science and Engineering Tsinghua University Beijing 100084 P. R. China; ^4^ Department of Physics Nanjing University Nanjing 210023 P. R. China; ^5^ National Laboratory of Solid State Microstructures Jiangsu Provincial Key Laboratory for Nanotechnology Collaborative Innovation Center of Advanced Microstructures and Department of Physics Nanjing University Nanjing 210023 P. R. China

**Keywords:** antiferromagnets, spin currents, spin dynamics, terahertz spectroscopy

## Abstract

An important vision of modern magnetic research is to use antiferromagnets (AFMs) as controllable and active ultrafast components in spintronic devices. Hematite (*α*‐Fe_2_O_3_) is a promising model material in this respect because its pronounced Dzyaloshinskii‐Moriya interaction leads to the coexistence of antiferromagnetism and weak ferromagnetism. Here, femtosecond laser pulses are used to drive terahertz (THz) spin currents from *α*‐Fe_2_O_3_ into an adjacent Pt layer. Two contributions to the generation of the spin current with distinctly different dynamics are found: the impulsive stimulated Raman scatting that relies on the AFM order and the ultrafast spin Seebeck effect that relies on the net magnetization. The total THz spin current dynamics can be manipulated by a medium‐strength magnetic field below 1 T. The control of the THz spin current achieved in *α*‐Fe_2_O_3_ opens the pathway toward tailoring the exact spin current dynamics from ultrafast AFM spin sources.

## Graphical abstract

An important vision of modern magnetic research is to use antiferromagnets (AFMs) as controllable and active ultrafast components in spintronic devices. The total terahertz (THz) spin current dynamics in hematite are manipulated by a medium‐strength magnetic field. The control of the THz spin current opens the pathway toward tailoring the exact spin current dynamics from ultrafast AFM spin sources.

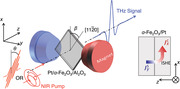

## 1 Introduction

Taming antiferromagnetism is a great challenge for modern magnetism research.^[^ ^1^, ^2^, ^3^, ^4^, ^5^ ^]^ Though notoriously difficult to manipulate,^[^ ^6^, ^7^, ^8^ ^]^ antiferromagnets (AFMs) keep fascinating researchers because of their stability against an external magnetic perturbation and the potential for ultrafast operations in the terahertz (THz) frequency range.^[^ ^9^, ^10^, ^11^, ^12^, ^13^ ^]^ In recent years, miscellaneous experimental strategies, e.g., THz‐driven linear^[^ ^14^, ^15^, ^16^, ^17^ ^]^ and nonlinear magnon responses,^[^ ^18^, ^19^ ^]^ THz magnon‐phonon coupling,^[^ ^20^, ^21^, ^22^, ^23^ ^]^ and THz magnetoelectric coupling,^[^ ^24^, ^25^ ^]^ provided deep insights into the ultrafast response of AFMs. As an emerging phenomenon, AFM spin pumping without the need for a strong external magnetic field is realized by the use of laser‐induced THz spin currents.^[^ ^26^, ^27^ ^]^ It prospectively devises a feasible scheme for practical antiferromagnetic spintronic devices, and more efforts on this topic are urgently required.

Hematite (*α*‐Fe_2_O_3_) is ubiquitous on earth, and its antiferromagnetic properties have been long studied.^[^ ^28^, ^29^, ^30^, ^31^ ^]^ It belongs to the trigonal crystal system, and the two magnetic sublattices antiferromagnetically align within the basal plane (0001) between the Morin temperature *T*_M_ ≈ 260 K and Néel temperature *T*_N_ ≈ 960 K. The presence of the Dzyaloshinskii‐Moriya interaction (DMI), described by the antisymmetric term in the exchange interaction Hamiltonian, gives rise to a small net magnetization ***M*** by slightly canting the two spin sublattices.^[^ ^32^, ^33^ ^]^ A relatively low spin‐flop field (<1 T) can align the Néel vector ***L*** perpendicular to the external field direction.^[^ ^30^ ^]^ The response of *α*‐Fe_2_O_3_ to moderate magnetic fields makes it a widely studied antiferromagnetic material in spintronics.^[^ ^34^, ^35^, ^36^ ^]^ Recently, the dc spin pumping by the acoustic resonant mode in *α*‐Fe_2_O_3_ enhanced by the DMI was reported.^[^ ^37^ ^]^ However, whether *α*‐Fe_2_O_3_ can generate THz spin currents upon ultrafast laser excitation remains an open question.

In this paper, we present the coexistence of two mechanisms for the generation of THz spin currents in *α*‐Fe_2_O_3_. At zero magnetic fields, antiferromagnetic spin pumping by an impulsive stimulated Raman scattering process initiates the injection of spin momentum from the *α*‐Fe_2_O_3_ layer to an adjacent Pt layer. This spin current can be superimposed by a considerable contribution of the ultrafast spin Seebeck effect when applying an external magnetic field as a direct consequence of the DMI. This tunability of the THz spin current polarity and dynamics achieved in *α*‐Fe_2_O_3_ by an external magnetic field provides more flexibility for high‐speed antiferromagnetic spintronic devices.

## 2 Results and Analysis

### 2.1 Experimental Geometry

**Figure 1:**
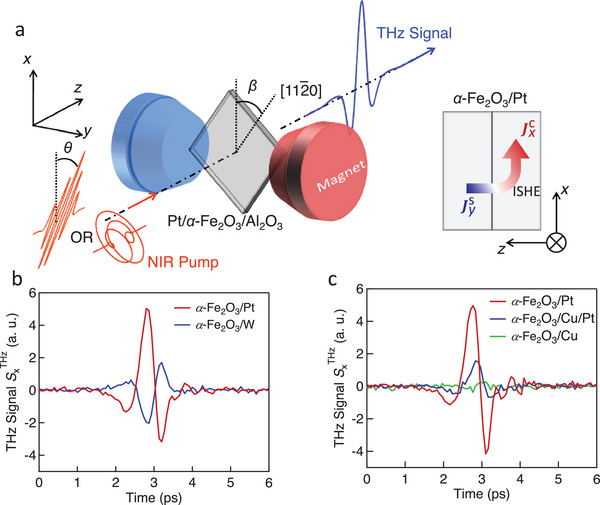
THz signals from the *α*‐Fe_2_O_3_/HM structures. a) Schematic of the transmission‐type THz spectroscopy setup. The coordinate (***xyz***) is adapted to the laboratory frame. The sample is placed in the *x*‐*y* plane and the laser is incident along the *z*‐direction. The magnetic field ***B***_ext_ is applied along ***y*** (as shown in panel (a)) or ***x*** (not shown). The polarization angle of the pump laser and the rotation angle of the sample are defined as *θ* and *β*, respectively. The sketch of the injection of the spin current $J_{y}^{s}$ from *α*‐Fe_2_O_3_ to the Pt layer is illustrated on the right. The spin current polarized along ***y*** is converted to the charge current $J_{x}^{c}$ along ***x***. b) THz signal $S_{x}^{\mathrm{THz}}$ of the *α*‐Fe_2_O_3_/Pt sample for ***B***_ext_ = 0. The polarities of THz signals from *α*‐Fe_2_O_3_/Pt (red) and *α*‐Fe_2_O_3_/W (blue) are opposite. c) THz signal from the *α*‐Fe_2_O_3_/Cu/Pt (blue) is weaker than that from *α*‐Fe_2_O_3_/Pt (red). The THz signal from *α*‐Fe_2_O_3_/Cu (green) is negligible. *θ*, *β* = 0° in (b and c). a. u., arbitrary units.

**Figure** **1**a illustrates the measurement scheme of the transmission‐type THz emission spectroscopy (Experimental Section). The coordinate system (***xyz***) is defined in the laboratory frame. The linearly or circularly polarized pump laser is incident along the *z*‐axis. For linear laser polarization, the polarization direction is denoted by *θ*. The 20‐nm thick (0001)‐oriented *α*‐Fe_2_O_3_ is grown on the Al_2_O_3_ substrate and the capping heavy metal (HM) Pt (thickness of 3 nm) is grown in situ (Experimental Section and Section S1, Supporting Information). The samples are placed in an external magnetic field ***B***_ext_ that is perpendicular to the *z*‐axis and in the sample plane. The angle between the $\left[11\bar{2}0\right]$ axis and the *x*‐axis is referred to as *β*. The laser‐induced THz signal from the samples propagates along the *z*‐axis and is probed via the linear electro‐optic effect in a 1‐mm‐thick (110)‐oriented ZnTe crystal (Experimental Section).

The red curve in Figure 1b shows a typical waveform of the THz signal $S_{x}^{\mathrm{THz}}$ (*x*‐component of the THz electric field) from *α*‐Fe_2_O_3_/Pt obtained with conditions *θ* = 0°, *β* = 0°, and ***B***_ext_ = 0. The THz spectrum covers the range from 0 to 3 THz (Section S2, Supporting Information). $S_{x}^{\mathrm{THz}}$ is confirmed to be linearly polarized by checking the two orthogonal electric components with two combined wire grid polarizers. The THz signal amplitude has a linear relationship with the laser fluence within the pulse‐energy range of interest (Section S3, Supporting Information).

### 2.2 Terahertz (THz) Spin Current in the *α*‐Fe_2_O_3_/Pt Bilayer

To reveal the origin of $S_{x}^{\mathrm{THz}}$, we prepared control samples *α*‐Fe_2_O_3_, *α*‐Fe_2_O_3_/W, *α*‐Fe_2_O_3_/Cu, and *α*‐Fe_2_O_3_/Cu/Pt. In Figure 1b, the polarity of $S_{x}^{\mathrm{THz}}$ from *α*‐Fe_2_O_3_/Pt is opposite to that from *α*‐Fe_2_O_3_/W. It agrees well with the opposite spin Hall angles of Pt and W.^[^ ^38^ ^]^ A 3‐nm‐thick Cu interlayer attenuates $S_{x}^{\mathrm{THz}}$ of *α*‐Fe_2_O_3_/Cu/Pt to less than half of that of *α*‐Fe_2_O_3_/Pt (Figure 1c). It is explained as that spin current flows through the Cu layer from the *α*‐Fe_2_O_3_ into the Pt layer and undergoes losses during transmission.^[^ ^26^, ^39^ ^]^ The THz signals from the bare *α*‐Fe_2_O_3_ film and the *α*‐Fe_2_O_3_/Cu structure are much smaller, indicating the HM layer is indispensable for a strong THz emission.

The aforementioned standard tests confirm the flow of an ultrafast spin current in Pt and support the following scenario: The spin current ***J*** ^s^(*t*) is injected from the *α*‐Fe_2_O_3_ layer into the Pt layer, as illustrated by the schematic on the right‐hand side of Figure 1a; ***J*** ^s^(*t*) is converted into an in‐plane charge current ***J*** ^c^(*t*) in the Pt layer by the inverse spin Hall effect.^[^ ^40^ ^]^ The transient ***J*** ^c^(*t*) emits a THz wave $S_{x}^{\mathrm{THz}}$ into free space. The THz spin current polarized along ***y*** ($J_{y}^{s}$) can be retrieved from $S_{x}^{\mathrm{THz}}$ by taking advantage of the measured response function of the THz emission setup (Experimental Section). In the following, we focus on $J_{y}^{s}$.

### 2.3 Opto‐Magnetic Origin of the THz Spin Current at Zero Magnetic Fields

Generally, the ultrafast spin injection can be realized by the incoherent driving forces: pyrospintronic effect (PSE)^[^ ^41^, ^42^, ^43^ ^]^ and ultrafast spin Seebeck effect (SSE)^[^ ^39^, ^42^, ^44^ ^]^ and the coherent driving forces: impulsive stimulated Raman scattering (ISRS)^[^ ^26^, ^27^ ^]^ and strain‐wave mediated magneto‐elastic coupling.^[^ ^27^ ^]^ The incoherent driving forces PSE and SSE are heating‐induced spin‐voltage and temperature gradient across the AFM/HM interface, respectively. They require non‐zero preexisting net magnetization in the magnetic layer. In contrast, the coherent driving forces induce impulsive magnetization in the AFM layer and pump the spin current into the HM layer, wherein preexisting net magnetization is not mandatory.

At room temperature, the as‐grown *α*‐Fe_2_O_3_ film is expected to contain magnetic domains orienting randomly along all easy axes ($\left\langle 1\bar{1}00\right\rangle$ axes). The grain size of each single spin domain *σ* is of the order of 1 µm.^[^ ^45^ ^]^ Therefore, within the area of the laser spot (diameter of ≈3 mm), the magnetization $\sum_{\sigma }M$ summed over all domains *σ* is approximately zero. We, thus, ascribe the generation of $J_{y}^{s}$ to ultrafast spin pumping launched by the laser‐induced transient magnetization Δ ***M*** (*t*).^[^ ^26^, ^27^, ^46^, ^47^ ^]^ The dynamic Δ ***M*** (*t*) originates from an effective magnetic field that is induced by the optical field through an ISRS process, as observed in AFMs previously.^[^ ^48^, ^49^, ^50^ ^]^

**Figure 2:**
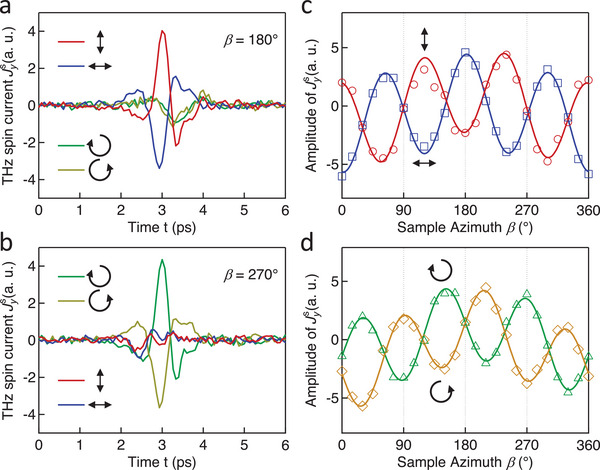
Impact of the state of the laser polarization at $\sum_{\sigma }M=\;0$. The traces of $J_{y}^{s}$ for various laser polarization states at (a) *β* = 180° and (b) *β* = 270°. The amplitude of $J_{y}^{s}$ varies (c) as a slightly distorted cos(3*β*) function when using the linear pump polarization and (d) as a slightly distorted sin(3*β*) function when using the circular pump polarization. The marks are measured results and the solid curves are fit. Laser polarization state: Red, along ***x***; Blue, along ***y***; Green, left; Yellow, right.

An off‐resonant and coherent ISRS response is expected to sensitively depend on the pump polarization and is, thus, tested by studying the impact of the pump polarization on $J_{y}^{s}$. As shown in **Figure** **2**a, at a sample azimuth of *β* = 180°, a linearly polarized pump beam is more efficient in generating $J_{y}^{s}$ than a circularly polarized one. In addition, the polarity of the traces is reversed for linear polarization directions *θ* = 0° (red) and *θ* = 90° (blue). In contrast, at *β* = 270°, a circularly rather than a linearly polarized pump facilitates the generation of $J_{y}^{s}$ (Figure 2b). The reversed sign of the current traces is compelling evidence for the pump‐helicity dependence.

Note that both Figure 2a,b indicates a relatively small $J_{y}^{s}$ component with minor dependence on the pump polarization. We ascribe this polarization‐insensitive contribution to the strain wave generated in the heating process of the Pt layer as observed previously.^[^ ^27^, ^30^, ^51^, ^52^ ^]^ The slightly different dynamics for the polarization‐insensitive contributions at different *β* might indicate an anisotropic magnetoelastic coupling and requires further investigation. Importantly, the strong dependence of the dominant $J_{y}^{s}$ contributions on the laser polarization state is a major indication for the ISRS process.^[^ ^49^, ^53^ ^]^

In principle, the off‐resonant $J_{y}^{s}$ component can be launched through ISRS that depends on the magnetic order parameter in first order (inverse Faraday effect (IFE)) or second order (inverse Cotton‐Mouton effect (ICME)).^[^ ^49^ ^]^ Interestingly, Figure 2a,b implies that ICME and IFE dominate for complementary orthogonal sample azimuths *β*. The dependence of the amplitude of $J_{y}^{s}$ on *β* is compared in detail for the cases of ICME (Figure 2c) and IFE (Figure 2d). The threefold period in all curves is consistent with the trigonal symmetry of the (0001)‐oriented *α*‐Fe_2_O_3_ film. Notably, $J_{y}^{s}$ associated with the ICME varies largely as a cos(3*β*) function, while that associated with IFE varies largely as a sin(3*β*) function. This pump‐polarization dependence is also observed in *α*‐Fe_2_O_3_/W (Section S4, Supporting Information). The reason is that the polarization of the spin current generated by the linearly polarized pump is mainly determined by ***M***, while that generated by the circularly polarized pump is mainly determined by ***L*** (Experimental Section). The slight distortion of the curves in Figure 2c,d is attributed to the lateral inhomogeneity of our *α*‐Fe_2_O_3_ films.

In summary, we can denote the THz spin current observed at zero magnetic fields as $J_{y}^{s,\mathrm{ISRS}}$, with a superscript indicating the ISRS origin. It can be phenomenologically described as the temporal convolution of the opto‐magnetic coefficients for the ISRS and the laser fluence^[^ ^26^ ^]^

(1) $$\begin{array}{ccc} J_{y}^{s,\mathrm{ISRS}}\left(t\right) & = & \sum_{\sigma }(\chi_{yii}^{\mathrm{lin}}\left(M_{x^{\prime }}\right)*E_{i}E_{i}^{*}+\chi_{y}^{\mathrm{cir}}\left(L_{y^{\prime }}\right)\\ & & *\left(E_{x}E_{y}^{*}-E_{x}^{*}E_{y}\right))\;\left(t\right) \end{array}$$

with ***E*** being the electric field of the pump laser pulse. The opto‐magnetic coefficients $\chi_{yii}^{\mathrm{lin}}$ (*i* = *x* or *y*) and $\chi_{y}^{\mathrm{cir}}$ are obtained by transforming the local coefficients in the spin coordinate (***x*** ′ ***y*** ′ ***z*** ′) to the lab coordinate (***xyz***). The summation convention for repeated indices is applied on the $\chi_{yii}^{\mathrm{lin}}$ term. The total spin current is obtained by summing the contribution from each magnetic domain *σ*. The fit (solid curves in Figure 2c,d) based on Equation 1 has a quantitative agreement with the experimental data (marks).

### 2.4 Manipulation of the THz Spin Current by an External Magnetic Field

**Figure 3:**
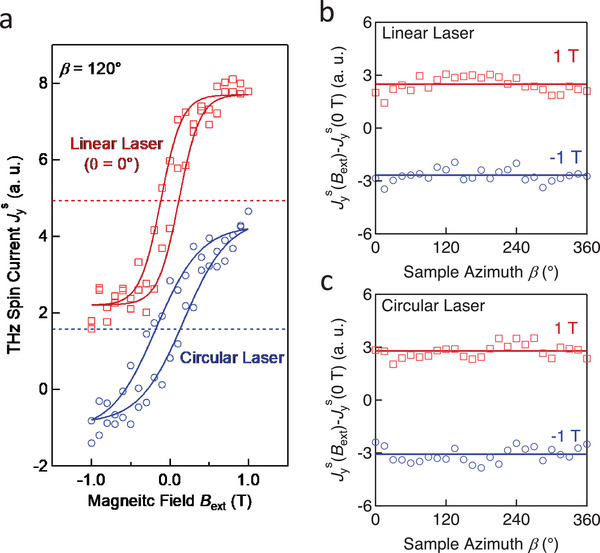
Manipulation of $J_{y}^{s}$ by ***B***_ext_. a) Amplitude of $J_{y}^{s}$ versus ***B***_ext_∥ ***y*** for linearly (red) and circularly (blue) polarized pump pulses and *β* = 120°. The solid curves are sigmoid fits. b) The additional contribution to $J_{y}^{s}$ while applying | ***B***_ext_| = 1 T is extracted by taking $J_{y}^{s}\left(B_{\mathrm{ext}}\right)-J_{y}^{s}\left(0\;T\right)$. A linear pump polarization (*θ* = 0) is used in the measurement. c) Analogous to panel (b) for circular pump polarization. The solid lines indicate the average values of $J_{y}^{s}$ when ***B***_ext_ is 1 T (red) or ‐1 T (blue). Note that the vertical offset of all data is as measured.

A non‐zero net magnetization $\sum_{\sigma }M\neq 0$ appears when an external magnetic field is applied to the *α*‐Fe_2_O_3_ film. We study the influence of $\sum_{\sigma }M\neq 0$ on $J_{y}^{s}$ by scanning ***B***_ext_∥ ***y*** in the range from −1 T to 1 T. As seen in **Figure** **3**a, $J_{y}^{s}$ exhibits a hysteretic feature in both measurements conducted with linearly (red) and circularly (blue) polarized laser pulses, which is in stark contrast to the linear magnetic response of KCoF_3_/Pt and KNiF_3_/Pt structures.^[^ ^54^ ^]^ The solid curves are a sigmoid fit to the experimental data, yielding a coercivity field lower than 0.15 T (Section S5, Supporting Information). The slight deviation between the two hysteresis loops is probably caused by a lateral shift of the sample position between the two measurements. The hysteretic response of $J_{y}^{s}$ is highly consistent with magnetic‐moment measurements via a superconducting quantum interface device (SQUID) at 300 K (Section S1, Supporting Information). We, thus, conclude that there is an additional contribution to $J_{y}^{s}$ by the nonzero net magnetization $\sum_{\sigma }M\neq 0$.

Note that two hysteresis loops in Figure 3a exhibit as‐measured vertical offsets. (The hysteresis loops for various *β* are shown in Section S5, Supporting Information.) The horizontal dashed line in each loop shows the average value during the fitting, which is close to the amplitude of $J_{y}^{s,\mathrm{ISRS}}$ that is obtained for $\sum_{\sigma }M=\;0$ (Figure 2c). Thus, the additional contribution to $J_{y}^{s}$ by the nonzero net magnetization $\sum_{\sigma }M\neq 0$ is superimposed on $J_{y}^{s,\mathrm{ISRS}}$ as a constant part that does not change with pump polarization or sample azimuth.

As shown in Figure 3a, the polarization state (linear or circular) of the pump laser does not affect the amplitude of the additional spin‐current contribution for $\sum_{\sigma }M\neq 0$. For better comparison, we extract the additional contribution by calculating the difference $J_{y}^{s}\left(B_{\mathrm{ext}}\right)-J_{y}^{s}\left(0\;T\right)$ for various *β* when ***B***_ext_ is kept at 1 T or ‐1 T. Both for using linear (Figure 3b) and circular (Figure 3c) pump polarization, $J_{y}^{s}$ contains a large *β*‐independent offset. Similar results can also be observed by studying the dependence of $J_{y}^{s}$ on *θ* (Section S6, Supporting Information). The minor fluctuation is probably attributed to the small changes in the opto‐magnetic coefficient for the ISRS under the influence of ***B***_ext_.^[^ ^53^ ^]^ The sizeable *β*‐independent part, as denoted by the solid horizontal line in Figure 3b,c, is odd in ***B***_ext_∥ ***y*** and not impacted by the state of the pump pulse polarization. This observation indicates the emergence of a spin‐current contribution for $\sum_{\sigma }M\neq 0$ in addition to the ultrafast off‐resonant contribution that dominates at $\sum_{\sigma }M=\;0$.

### 2.5 Spin‐Caloritronic Contribution to the THz Spin Current at a Finite External Magnetic Field

Coherent and incoherent driving forces exhibit different temporal evolution of the ultrafast spin current. Therefore, the time scale of the ultrafast spin current evolution can be used as the hallmark to clarify its origin.^[^ ^42^ ^]^ To capture the ultrafast evolution of $J_{y}^{s}$, we conduct a measurement in the THz emission setup based on a 15‐fs Ti:sapphire laser oscillator (Experimental Section).

**Figure 4:**
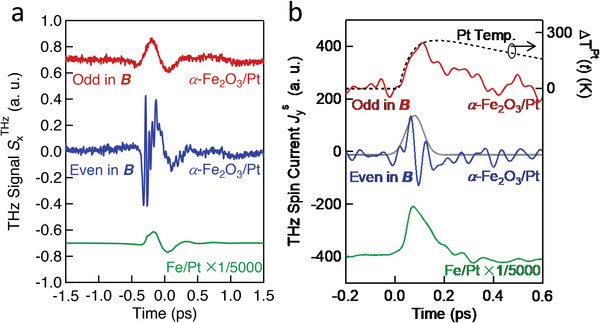
Temporal evolution of $J_{y}^{s}$ in *α*‐Fe_2_O_3_/Pt and Fe/Pt. a) Component of the THz signal $S_{x}^{\mathrm{THz}}$ that is odd (red) and even (blue) in the external magnetic field ***B***_ext_ = 0.4 T. The green curve shows the evolution of $J_{y}^{s}$ in Fe/Pt as a comparison. b) The spin current $J_{y}^{s}$ retrieved from (a). Compared with Fe/Pt (green), the $J_{y}^{s}$ component odd in the external magnetic field (red) in *α*‐Fe_2_O_3_/Pt slowly evolves with a rise time >100 fs, whereas the fast even component (blue) has an impulsive feature, basically following the pump‐pulse intensity envelope superimposed by a fast oscillatory signal. The dotted curve is the calculated generalized electronic temperature in Pt following laser excitation (Adapted from^[^ ^39^ ^]^). The grey curve is a Gaussian fit with a full width at half maximum of ≈50 fs. Note that the curves in (b) were smoothed with a Gaussian function with a 1/e width of 26 fs for better visibility. The curves are vertically shifted for clarity.

As shown in **Figure** **4**a, the odd (red) and even (blue) components of the THz signal $S_{x}^{\mathrm{THz}}$ in the magnetic field are extracted by taking the difference $S_{x}^{\mathrm{THz}}\left(B_{\mathrm{ext}}\right)-S_{x}^{\mathrm{THz}}\left(-B_{\mathrm{ext}}\right)$ and the sum $S_{x}^{\mathrm{THz}}\left(B_{\mathrm{ext}}\right)+S_{x}^{\mathrm{THz}}\left(-B_{\mathrm{ext}}\right)$, respectively. The magnetic field ***B***_ext_ = 0.4 T is higher than the spin‐flop field of our *α*‐Fe_2_O_3_ film (Section S1, Supporting Information). The odd THz signal of a fully metallic Fe/Pt thin‐film structure (green) is shown for comparison. The fast‐even component has an impulsive feature, basically following the pump‐pulse intensity envelope superimposed by a fast oscillatory signal. The high‐frequency oscillation is attributed to the excitation of phonon modes in *α*‐Fe_2_O_3_ within the frequency range from 10 to 20 THz.^[^ ^48^, ^55^, ^56^ ^]^ Its detailed origin requires further studies.

The waveforms of spin current $J_{y}^{s}$ are retrieved from Figure 4a and correspondingly shown in Figure 4b. The high‐frequency oscillation is removed in Figure 4b by smoothening the curve with a Gaussian function with a 1/e‐width of 26 fs. The even component of $J_{y}^{s}$ (blue) is largely $J_{y}^{s,\mathrm{ISRS}}$. In principle, the even component could include nonmagnetic signal contributions, which we, however, assume to be minor due to the absence of any detectable THz emission in the *α*‐Fe_2_O_3_/Cu control sample (Figure 1c). As a reference, we compare our extracted spin currents to that of a fully metallic Fe/Pt thin‐film structure (green), which has a relatively rapid rise and decay. The driving force in Fe/Pt is the PSE, and the time scale of its relaxation is mostly determined by the electron‐spin equilibration time in the ferromagnetic metal.^[^ ^43^ ^]^ In contrast, the temporal evolution of the odd component of $J_{y}^{s}$ (red) in the *α*‐Fe_2_O_3_/Pt structure exhibits a slower rise time of more than 100 fs and an even slower decay. These markedly different time scales strongly suggest driving forces different from the ISRS process^[^ ^27^ ^]^ and the PSE and rather indicate that the odd component of $J_{y}^{s}$ is dominated by the ultrafast SSE.

A temperature gradient across the interface of the magnetic and the paramagnetic layer is necessary for the ultrafast SSE. In the case of a magnetic insulator, laser‐excited hot electrons in the metal layer (Pt) get spin‐polarized upon scattering off the interface toward the magnetic insulator.^[^ ^57^ ^]^ In *α*‐Fe_2_O_3_/Pt, the electronic temperature of the Pt layer increases by Δ*T* ^Pt^(t) upon laser excitation,^[^ ^58^, ^59^ ^]^ while that of the *α*‐Fe_2_O_3_ layer remains unaffected because of the weak absorption.^[^ ^60^ ^]^ Therefore, the ultrafast‐SSE spin current injected from the *α*‐Fe_2_O_3_ to the Pt layer can be expressed as the temporal convolution $J_{y}^{s,\Delta T}\;\left(t\right)=\left(\kappa_{y}^{\mathrm{Pt}}*\Delta T^{\mathrm{Pt}}\right)\;\left(t\right)$, with $\kappa_{y}^{\mathrm{Pt}}\left(t\right)$ being the response function that relates the spin current in the Pt layer to an ultrashort *δ*‐like temperature increase of the Pt electrons. This response function is proportional to the convolution of the spin susceptibilities of the Pt and the *α*‐Fe_2_O_3_ layer.

### 2.6 Quasi‐instantaneous Fit to the Spin‐Seebeck Spin Current

While Δ*T* ^Pt^(*t*) has an ultrafast rise and a decay time on the order of 100 fs, $\kappa_{y}^{\mathrm{Pt}}\left(t\right)$ has a much shorter duration and is dominated by the spin‐spin correlation time in Pt, which is of the order of a few femtoseconds only.^[^ ^39^ ^]^ As a result, $\kappa_{y}^{\mathrm{Pt}}$ acts like a *δ*‐like function, and $J_{y}^{s,\Delta T}$ follows Δ*T* ^Pt^(*t*) quasi‐instantaneously:

(2) $$J_{y}^{s,\Delta T}=\;\eta \left(\sum_{\sigma }M_{y}\right)\Delta T^{\mathrm{Pt}}\left(t\right)$$

The SSE coefficient *η* is odd in the net magnetization and, thus, scales linearly with $\sum_{\sigma }M_{y}$ to lowest order. As excited by the ultrafast laser pulses, highly energetic electrons in Pt are generated. Subsequently, the electrons thermalize via secondary scattering cascades to form more carriers above the Fermi energy yet with individually less energy. The calculated temperature evolution Δ*T* ^Pt^(*t*) for the electrons in Pt is shown by the dashed curve in Figure 4b (Experimental Section). Based on these considerations, we can understand the apparently good agreement between Δ*T* ^Pt^(*t*) and $J_{y}^{s,\Delta T}$.

One should, however, note that the rise time of the spin current in YIG/Pt, *γ*‐Fe_2_O_3_/Pt, and Fe_3_O_4_/Pt structures^[^ ^42^ ^]^ is ≈100 fs slower than that in *α*‐Fe_2_O_3_/Pt. We speculate that these discrepancies can arise from altered thermalization dynamics in Pt layers grown under different conditions on different magnets (electron scattering times might alter significantly). Alternatively, direct excitation of carriers in *α*‐Fe_2_O_3_ due to its relatively small band gap of ≈2.1 eV in comparison to the pump photon energy of 1.5 eV may occur.^[^ ^61^ ^]^ The latter scenario might lead to a spin‐voltage‐like driving force that entails a contribution with faster dynamics than the typical SSE current measured in previous experiments. Indeed, we find that the measured spin current odd in magnetization in *α*‐Fe_2_O_3_/Pt can be reproduced by a linear combination of the spin current driven by the ultrafast spin voltage in Fe/Pt and the ultrafast Seebeck current in a YIG/Pt sample (Section S7, Supporting Information).

## 3 Discussion

Our analysis indicates that the total THz spin current can be rewritten as $J_{y}^{s}=J_{y}^{s,\mathrm{ISRS}}\;+J_{y}^{s,\Delta T}$. The insensitivity of the laser‐induced Δ*T^Pt^* (t) to the laser polarization^[^ ^39^, ^62^ ^]^ is in line with the *β*‐independent contribution shown in Figure 3a,b. Besides, according to Equation 2, $\sum_{\sigma }M∥x$ does not contribute to $J_{y}^{s}$ via the ultrafast SSE. As a test, the dependence of $J_{y}^{s}$ on *β* is measured when ***B***_ext_∥ ***x*** (1 T) is applied (Section S8, Supporting Information). The absence of the *β*‐independent modulation of $J_{y}^{s}$ by ***B***_ext_∥ ***x*** fully agrees with our interpretation including the ultrafast SSE.

Multiple spin current generation mechanisms can coexist in magnetic systems. The laser‐excited electrons carrying spin angular momentum in metallic systems can inject a spin current driven by the spin voltage. It is overwhelmingly predominant in ferromagnetic metals and largely outperforms other mechanisms. Therefore, the insulating antiferromagnetic *α*‐Fe_2_O_3_ provides a platform to observe the ultrafast coherent spin pumping and the ultrafast SSE simultaneously. In contrast, in NiO/Pt structures, there is no obvious THz spin current driven by the ultrafast SSE because of the absence of a net magnetization due to a lack of DMI (Section S9, Supporting Information).

Hematite is classified as a g‐wave altermagnet with four nodal surfaces in the Brillouin zone.^[^ ^63^, ^64^ ^]^ Because g‐wave altermagnets have a spin‐independent averaged electrical conductivity, the contribution to the giant magnetoresistance from the spin‐dependent averaged conductivities is absent.^[^ ^65^, ^66^ ^]^ Analogously, we expect no altermagnetism‐related contributions to the SSE, assuming that the thermal spin transport follows the same symmetry rules as the electrical spin transport.

The laser‐induced Morin transition can give rise to a spin reorientation in iron oxides.^[^ ^9^ ^]^ For bulk *α*‐Fe_2_O_3_, the Morin temperature is ≈260 K and it considerably decreases for thin films.^[^ ^67^, ^68^ ^]^ Our measurements were conducted at room temperature (≈300 K), which is much higher than the actual Morin temperature. As a result, there is no laser‐induced spin reorientation in our experiments.

The even component of $J_{y}^{s}$ (blue) obtained by the sum $J_{y}^{s}\left(0.4\;T\right)+J_{y}^{s}\left(-0.4\;T\right)$ shows an impulsive response, that is, it follows the intensity envelope of the pump pulse. The grey curve in Figure 4b is a Gaussian fit with a full width at half maximum of ≈50 fs, which combines the pump pulse duration (15 fs) and the bandwidth of the current extraction procedure (1/30 THz = 33 fs) as well as the intrinsic time scale of the ISRS. The latter was calculated to have dynamics of the order of 30 fs,^[^ ^69^ ^]^ which agrees well with our findings.

## 4 Conclusion

In summary, we comprehensively studied the mechanisms for the generation of the THz spin current $J_{y}^{s}$ in the *α*‐Fe_2_O_3_/Pt structure. The presence of DMI causes weak spontaneous magnetization in spin domains of *α*‐Fe_2_O_3_. At $\sum_{\sigma }M=\;0$, the ultrafast spin pumping is facilitated by the ISRS process and dominates the generation of $J_{y}^{s}$. ICME and IFE predominate in orthogonal directions. The ultrafast ISRS‐mediated spin pumping acts on a time scale of a few tens of femtoseconds. The ultimate time scale may be still faster because of the finite laser pulse duration. At $\sum_{\sigma }M\neq 0$, the ultrafast SSE additionally contributes to the generation of $J_{y}^{s}$, which evolves slowly with respect to that generated via ultrafast spin pumping. Our results are important in understanding the origin of the THz response in AFM/HM structures, which received much interest recently. Future studies based on time‐resolved magneto‐optical Kerr effect or temperature‐dependent measurements may provide further insights. From an applied viewpoint, the spin current related to the Néel vector and the net magnetization can be distinguished by checking the dependence on the laser polarization, which demonstrates a new technical scheme for detecting the detailed spin texture in AFM even on ultrafast time scales. More importantly, the adjustable net magnetization opens an exciting pathway toward the control of the exact spin current dynamics from ultrafast AFM spin sources.

## 5 Experimental Section

### Measurement

All measurements except that shown in Figure 4 were conducted with a THz‐emission setup based on a Ti:sapphire amplified laser. The laser provided pulses with 100‐fs duration, 1‐kHz repetition rate, and 800‐nm central wavelength. The pump beam was loosely focused on the sample surface with a spot diameter of ≈3 mm. The laser fluence was ≈1.2 mJ cm^−2^.

In Figure 4, the evolution of the spin current was measured with a 15‐fs Ti:sapphire laser oscillator (center wavelength 800 nm, pulse energy 2.5 nJ, and repetition rate 80 MHz). The duration of the laser pulses was compressed by using a pair of wedged prisms and a pair of chirped mirrors. The diameter of the pump beam at the sample surface was ≈20 µm. The resulting absorbed fluence was 0.12 mJ cm^−2^.

The magnetic field (| ***B***_ext_| ≤ 1 T) was applied by a dc electromagnet. The magnetic field was monitored with a Gauss meter. The divergent THz signal was collected by a parabolic mirror with a reflected focal length (RFL) of 101.6 mm. Another parabolic mirror (RFL = 50.8 mm) focused the collimated THz signal onto the 1‐mm‐thick (110)‐oriented ZnTe crystal with a spot diameter of ≈300 µm. The ellipticity modulation of the probe beam that was tightly focused on the center of the THz spot was captured by a pair of balanced photodetectors for the acquisition of the THz signal. All the measurements were carried out at room temperature (≈300 K) and in the dry air or N_2_ atmosphere.

### Sample Fabrication

The *α*‐Fe_2_O_3_/Pt samples were grown in a high vacuum magnetron sputtering chamber with a base vacuum of 5 × 10^−5^ Pa. The thickness was 20 nm for the *α*‐Fe_2_O_3_ layer and 3 nm for the Pt layer. The *α*‐Fe_2_O_3_ films were grown on (0001)‐oriented Al_2_O_3_ substrates under an atmosphere (Ar:O_2_ = 10:1) with the temperature of substrates at 500 °C. The Pt layer was then in situ capped on the *α*‐Fe_2_O_3_ layer at room temperature. The qualities of films were characterized by X‐ray diffraction. All samples in the main text were fabricated on the (0001)‐oriented Al_2_O_3_ substrates.

### Macroscopic Theory for Ultrafast Spin Pumping

In this section, it was focused on analyzing the relationship between ***J*** ^s^ in Pt and dynamics of ***M*** and ***L*** in *α*‐Fe_2_O_3_. The Hamiltonian describing the interaction between the laser and the medium was a function of the dielectric tensor *ε*_*ij*_. The laser pulse modulates *ε*_*ij*_, acted as effective fields $H_{\mathrm{eff}}^{M}$ and $H_{\mathrm{eff}}^{L}$, which were expressed as the partial derivative of the interaction Hamiltonian to ***M*** and ***L***, respectively.^[^ ^70^ ^]^ Therefore, the ISRS process induced an impulsive magnetization Δ ***M*** (*t*) in the *α*‐Fe_2_O_3_ layer and gave rise to spin pumping on a time scale of sub‐picoseconds.

The spin pumping in *α*‐Fe_2_O_3_/Pt was described as^[^ ^37^ ^]^

(3) $$J^{s}=\;\hbar g_{\mathrm{mix}}M\times \frac{\partial M}{\partial t},$$

in which ℏ is reduced Planck constant and *g*_mix_ is the interfacial spin‐mixing conductance. Here, the Landau‐Lifshitz‐Gilbert equations were used to describe the dynamics of ***M*** and ***L*** ^[^ ^70^ ^]^

(4) $$\frac{\partial M}{\partial t}=\;-\gamma \left\{M\times H_{\mathrm{eff}}^{M}+L\times H_{\mathrm{eff}}^{L}\right\},$$

where *γ* is the gyromagnetic ratio. Combining Equations 3 and 4, the spin pumping can be written as

(5) $$J^{s}=\;-\hbar g_{\mathrm{mix}}\gamma \left[M\left(M\cdot H_{\mathrm{eff}}^{M}\right)-H_{\mathrm{eff}}^{M}\left(M\cdot M\right)+L\left(M\cdot H_{\mathrm{eff}}^{L}\right)\right]$$

In the experimental geometry, only the spin current polarized in the plane can be detected. A single spin domain was focused on first. By taking into account of ***L*** ≫ ***M***, the spin pumping in the form of was written

(6) $$\left[\begin{array}{c} J_{x^{\prime }}^{s}\\ J_{y^{\prime }}^{s} \end{array}\right]=\;-\hbar g_{\mathrm{mix}}\gamma \left[\begin{array}{c} M_{x^{\prime }}M\cdot H_{\mathrm{eff}}^{M}\\ L_{y^{\prime }}M\cdot H_{\mathrm{eff}}^{L} \end{array}\right],$$

with (***x*** ′ ***y*** ′ ***z*** ′) as the coordinate in the spin frame. $H_{\mathrm{eff}}^{M}$ and $H_{\mathrm{eff}}^{L}$ were the effective fields generated via IFE and ICME, respectively (Section S10, Supporting Information). As a result, IFE and ICME predominate in orthogonal directions. The striking agreement between the measurement and the theory strongly suggested that the origin of the spin pumping was directly related to the ISRS.

There were three easy axes in the basal plane of *α*‐Fe_2_O_3_. The contribution of all spin domains could be calculated by transforming the tensors in each spin frame into that of the laboratory frame.^[^ ^71^ ^]^ The final form of the formula could be expressed as the linear superposition of the cos(3*β*) and cos(*β*) functions,^[^ ^49^, ^71^ ^]^ in which all the Opto‐magnetic coefficients were subsumed into the fitting parameters in front of the trigonometric functions. The fitting parameters for the curves in Figure 2c,d were provided in Section S11, Supporting Information.

The THz spin current originating from the ISRS process could be essentially described as Equation 1 in the main text. Note that the opto‐magnetic coefficients $\chi_{yii}^{\mathrm{lin}}$ (*i* = *x* or *y*) or $\chi_{y}^{\mathrm{cir}}$ was not only determined by ***M***_*x*′_ or ***L***_*y*′_. The simplified form was adopted in the main text to denote that ICME and IFE predominate in orthogonal directions.

### Extraction of the THz Current

The current extraction for the *α*‐Fe_2_O_3_ sample was done by using a reference THz signal from a fully metallic Fe/Pt sample, for which the current was known from the previous studies.^[^ ^42^ ^]^ A matrix inversion procedure was then applied to deconvolute the detected electro‐optic signals by the setup response function in the time domain.^[^ ^39^ ^]^

## Conflict of Interest

The authors declare no conflict of interest.

## Supporting information

Supporting Information

## Data Availability

The data that support the findings of this study are available from the corresponding author upon reasonable request.

## References

[1] T. Jungwirth , X. Marti , P. Wadley , J. Wunderlich , Nat. Nanotechnol. 2016, 11, 231. 26936817 10.1038/nnano.2016.18

[2] J. Železný , P. Wadley , K. Olejník , A. Hoffmann , H. Ohno , Nat. Phys. 2018, 14, 220.

[3] O. Gomonay , V. Baltz , A. Brataas , Y. Tserkovnyak , Nat. Phys. 2018, 14, 213.

[4] V. Baltz , A. Manchon , M. Tsoi , T. Moriyama , T. Ono , Y. Tserkovnyak , Rev. Mod. Phys. 2018, 90, 015005.

[5] M. B. Jungfleisch , W. Zhang , A. Hoffmann , Phys. Lett. Sect. A Gen. At. Solid State Phys. 2018, 382, 865.

[6] S. Manz , M. Matsubara , T. Lottermoser , J. Büchi , A. Iyama , T. Kimura , D. Meier , M. Fiebig , Nat. Photonics 2016, 10, 653.

[7] J. Li , C. B. Wilson , R. Cheng , M. Lohmann , M. Kavand , W. Yuan , M. Aldosary , N. Agladze , P. Wei , M. S. Sherwin , J. Shi , Nature 2020, 578, 70. 31988510 10.1038/s41586-020-1950-4

[8] P. Vaidya , S. A. Morley , J. van Tol , Y. Liu , R. Cheng , A. Brataas , D. Lederman , E. del Barco , Science 2020, 368, 160. 32273462 10.1126/science.aaz4247

[9] A. V. Kimel , A. Kirilyuk , A. Tsvetkov , R. V. Pisarev , T. Rasing , Nature 2004, 429, 850. 15215858 10.1038/nature02659

[10] T. Kampfrath , K. Tanaka , K. a. Nelson , Nat. Photonics 2013, 7, 680.

[11] T. Satoh , R. Iida , T. Higuchi , M. Fiebig , T. Shimura , Nat. Photonics 2014, 9, 25.

[12] P. Němec , M. Fiebig , T. Kampfrath , A. V. Kimel , Nat. Phys. 2018, 14, 229.

[13] H. Bai , X. Zhou , Y. Zhou , X. Chen , Y. You , F. Pan , C. Song , J. Appl. Phys. 2020, 128, 210901.

[14] N. Kanda , T. Higuchi , H. Shimizu , K. Konishi , K. Yoshioka , M. Kuwata‐Gonokami , Nat. Commun. 2011, 2, 362. 21694710 10.1038/ncomms1366 PMC3156816

[15] N. Bhattacharjee , A. A. Sapozhnik , S. Y. Bodnar , V. Y. Grigorev , S. Y. Agustsson , J. Cao , D. Dominko , M. Obergfell , O. Gomonay , J. Sinova , M. Kläui , H. J. Elmers , M. Jourdan , J. Demsar , Phys. Rev. Lett. 2018, 120, 237201. 29932703 10.1103/PhysRevLett.120.237201

[16] T. Kampfrath , A. Sell , G. Klatt , A. Pashkin , S. Mährlein , T. Dekorsy , M. Wolf , M. Fiebig , A. Leitenstorfer , R. Huber , Nat. Photonics 2011, 5, 31.

[17] K. Yamaguchi , T. Kurihara , Y. Minami , M. Nakajima , T. Suemoto , Phys. Rev. Lett. 2013, 110, 137204. 23581366 10.1103/PhysRevLett.110.137204

[18] S. Baierl , M. Hohenleutner , T. Kampfrath , A. K. Zvezdin , A. V. Kimel , R. Huber , R. V. Mikhaylovskiy , Nat. Photonics 2016, 10, 715.

[19] S. Baierl , J. H. Mentink , M. Hohenleutner , L. Braun , T. M. Do , C. Lange , A. Sell , M. Fiebig , G. Woltersdorf , T. Kampfrath , R. Huber , Phys. Rev. Lett. 2016, 117, 197201. 27858446 10.1103/PhysRevLett.117.197201

[20] S. Wall , D. Prabhakaran , A. T. Boothroyd , A. Cavalleri , Phys. Rev. Lett. 2009, 103, 3. 10.1103/PhysRevLett.103.097402 19792828

[21] T. F. Nova , A. Cartella , A. Cantaluppi , M. Först , D. Bossini , R. V. Mikhaylovskiy , A. V. Kimel , R. Merlin , A. Cavalleri , Nat. Phys. 2017, 13, 132.

[22] H. T. Simensen , R. E. Troncoso , A. Kamra , A. Brataas , Phys. Rev. B 2019, 99, 064421.

[23] K. W. Kim , A. Pashkin , H. Schäfer , M. Beyer , M. Porer , T. Wolf , C. Bernhard , J. Demsar , R. Huber , A. Leitenstorfer , Nat. Mater. 2012, 11, 497. 22484832 10.1038/nmat3294

[24] T. Kubacka , J. A. Johnson , M. C. Hoffmann , C. Vicario , S. de Jong , P. Beaud , S. Grübel , S.‐W. Huang , L. Huber , L. Patthey , Y.‐D. Chuang , J. J. Turner , G. L. Dakovski , W.‐S. Lee , M. P. Minitti , W. Schlotter , R. G. Moore , C. P. Hauri , S. M. Koohpayeh , V. Scagnoli , G. Ingold , S. L. Johnson , U. Staub , Science 2014, 343, 1333. 24603154 10.1126/science.1242862

[25] D. Bossini , K. Konishi , S. Toyoda , T. Arima , J. Yumoto , M. Kuwata‐Gonokami , Nat. Phys. 2018, 14, 370.

[26] H. Qiu , L. Zhou , C. Zhang , J. Wu , Y. Tian , S. Cheng , S. Mi , H. Zhao , Q. Zhang , D. Wu , B. Jin , J. Chen , P. Wu , Nat. Phys. 2021, 17, 388.

[27] E. Rongione , O. Gueckstock , M. Mattern , O. Gomonay , H. Meer , C. Schmitt , R. Ramos , E. Saitoh , J. Sinova , H. Jaffrès , M. Mičica , J. Mangeney , S. T. B. Goennenwein , S. Geprägs , T. Kampfrath , M. Kläui , M. Bargheer , T. S. Seifert , S. Dhillon , R. Lebrun , (Preprint) arXiv:2205.11965, v1, submitted: May 2022.

[28] C. G. Shull , W. A. Strauser , E. O. Wollan , Phys. Rev. 1951, 83, 333.

[29] Q. A. Pankhurst , C. E. Johnson , M. F. Thomas , J. Phys. C: Solid State Phys. 1986, 19, 7081.

[30] P. Zhang , J. Finley , T. Safi , L. Liu , Phys. Rev. Lett. 2019, 123, 247206. 31922833 10.1103/PhysRevLett.123.247206

[31] Y. Cheng , S. Yu , M. Zhu , J. Hwang , F. Yang , Phys. Rev. Lett. 2020, 124, 27202. 10.1103/PhysRevLett.124.027202 32004028

[32] I. Dzyaloshinsky , J. Phys. Chem. Solids 1958, 4, 241.

[33] T. Moriya , Phys. Rev. 1960, 120, 91.

[34] R. Lebrun , A. Ross , S. A. Bender , A. Qaiumzadeh , L. Baldrati , J. Cramer , A. Brataas , R. A. Duine , M. Kläui , Nature 2018, 561, 222. 30209370 10.1038/s41586-018-0490-7 PMC6485392

[35] L. Huang , Y. Zhou , H. Qiu , T. Guo , F. Pan , B. Jin , C. Song , Appl. Phys. Lett. 2021, 119, 212401.

[36] Y. Zhou , L. Liao , T. Guo , H. Bai , M. Zhao , C. Wan , L. Huang , L. Han , L. Qiao , Y. You , C. Chen , R. Chen , Z. Zhou , X. Han , F. Pan , C. Song , Nat. Commun. 2022, 13, 3723. 35764620 10.1038/s41467-022-31531-w PMC9240048

[37] H. Wang , Y. Xiao , M. Guo , E. Lee‐Wong , G. Q. Yan , R. Cheng , C. R. Du , Phys. Rev. Lett. 2021, 127, 117202. 34558931 10.1103/PhysRevLett.127.117202

[38] J. Sinova , S. O. Valenzuela , J. Wunderlich , C. H. Back , T. Jungwirth , Rev. Mod. Phys. 2015, 87, 1213.

[39] T. S. Seifert , S. Jaiswal , J. Barker , S. T. Weber , I. Razdolski , J. Cramer , O. Gueckstock , S. F. Maehrlein , L. Nadvornik , S. Watanabe , C. Ciccarelli , A. Melnikov , G. Jakob , M. Münzenberg , S. T. B. Goennenwein , G. Woltersdorf , B. Rethfeld , P. W. Brouwer , M. Wolf , M. Kläui , T. Kampfrath , Nat. Commun. 2018, 9, 2899. 30042421 10.1038/s41467-018-05135-2 PMC6057952

[40] E. Saitoh , M. Ueda , H. Miyajima , G. Tatara , Appl. Phys. Lett. 2006, 88, 182509.

[41] T. Kampfrath , M. Battiato , P. Maldonado , G. Eilers , J. Nötzold , S. Mährlein , V. Zbarsky , F. Freimuth , Y. Mokrousov , S. Blügel , M. Wolf , I. Radu , P. M. Oppeneer , M. Münzenberg , Nat. Nanotechnol. 2013, 8, 256. 23542903 10.1038/nnano.2013.43

[42] P. Jiménez‐Cavero , O. Gueckstock , L. Nádvorník , I. Lucas , T. S. Seifert , M. Wolf , R. Rouzegar , P. W. Brouwer , S. Becker , G. Jakob , M. Kläui , C. Guo , C. Wan , X. Han , Z. Jin , H. Zhao , D. Wu , L. Morellón , T. Kampfrath , Phys. Rev. B 2022, 105, 184408.

[43] R. Rouzegar , L. Brandt , L. Nadvornik , D. A. Reiss , A. L. Chekhov , O. Gueckstock , C. In , M. Wolf , T. S. Seifert , P. W. Brouwer , G. Woltersdorf , T. Kampfrath , Phys. Rev. B 2022, 106, 144427.

[44] A. Fognini , T. U. Michlmayr , A. Vaterlaus , Y. Acremann , J Phys Condens Matter 2017, 29, 214002. 28441145 10.1088/1361-648X/aa6a76

[45] F. P. Chmiel , N. Waterfield Price , R. D. Johnson , A. D. Lamirand , J. Schad , G. Van Der Laan , D. T. Harris , J. Irwin , M. S. Rzchowski , C. B. Eom , P. G. Radaelli , Nat. Mater. 2018, 17, 581. 29915425 10.1038/s41563-018-0101-x

[46] P. Stremoukhov , A. Safin , M. Logunov , S. Nikitov , A. Kirilyuk , J. Appl. Phys. 2019, 125, 223903.

[47] L. Bocklage , Phys. Rev. Lett. 2017, 118, 257202. 28696755 10.1103/PhysRevLett.118.257202

[48] R. V. Mikhaylovskiy , E. Hendry , A. Secchi , J. H. Mentink , M. Eckstein , A. Wu , R. V. Pisarev , V. V. Kruglyak , M. I. Katsnelson , T. Rasing , A. V. Kimel , Nat. Commun. 2015, 6, 8190. 26373688 10.1038/ncomms9190 PMC4595597

[49] C. Tzschaschel , K. Otani , R. Iida , T. Shimura , H. Ueda , S. Günther , M. Fiebig , T. Satoh , Phys. Rev. B 2017, 95, 174407.

[50] T. Satoh , R. Iida , T. Higuchi , Y. Fujii , A. Koreeda , H. Ueda , T. Shimura , K. Kuroda , V. I. Butrim , B. A. Ivanov , Nat. Commun. 2017, 8, 638. 28935962 10.1038/s41467-017-00616-2 PMC5608704

[51] L. Baldrati , C. Schmitt , O. Gomonay , R. Lebrun , R. Ramos , E. Saitoh , J. Sinova , M. Kläui , Phys. Rev. Lett. 2020, 125, 077201. 32857543 10.1103/PhysRevLett.125.077201

[52] S. Wust , C. Seibel , H. Meer , P. Herrgen , C. Schmitt , L. Baldrati , R. Ramos , T. Kikkawa , E. Saitoh , O. Gomonay , J. Sinova , Y. Mokrousov , H. C. Schneider , M. Kläui , B. Rethfeld , B. Stadtmüller , M. Aeschlimann , (Preprint) arXiv:2205.02686, v1, submitted: May 2022.

[53] K. Grishunin , E. A. Mashkovich , A. V. Kimel , A. M. Balbashov , A. K. Zvezdin , Phys. Rev. B 2021, 104, 024419.

[54] F. N. Kholid , D. Hamara , A. F. Bin Hamdan , G. N. Antonio , R. Bowen , D. Petit , R. Cowburn , R. V. Pisarev , D. Bossini , J. Barker , C. Ciccarelli , Nat. Commun. 2023, 14, 538. 36725847 10.1038/s41467-023-36166-z PMC9892507

[55] S. Onari , T. Arai , K. Kudo , Phys. Rev. B 1977, 16, 1717.

[56] C. Bacaksiz , M. Yagmurcukardes , F. M. Peeters , M. V. Milošević , 2D Mater. 2020, 7, 025029.

[57] H. Adachi , K. Uchida , E. Saitoh , S. Maekawa , Reports Prog. Phys. 2013, 76, 036501. 10.1088/0034-4885/76/3/036501 23420561

[58] D. Zahn , H. Seiler , Y. W. Windsor , R. Ernstorfer , Struct. Dyn. 2021, 8, 064301. 34805449 10.1063/4.0000120 PMC8594951

[59] V. Unikandanunni , F. Rigoni , M. C. Hoffmann , P. Vavassori , S. Urazhdin , S. Bonetti , Appl. Phys. Lett. 2022, 120, 021601.

[60] L. A. Marusak , R. Messier , W. B. White , J. Phys. Chem. Solids 1980, 41, 981.

[61] F. Paquin , J. Rivnay , A. Salleo , N. Stingelin , C. Silva , J. Mater. Chem. C 2015, 3, 10715.

[62] M. Schreier , A. Kamra , M. Weiler , J. Xiao , G. E. W. Bauer , R. Gross , S. T. B. Goennenwein , Phys. Rev. B 2013, 88, 094410.

[63] R. González‐Hernández , L. Šmejkal , K. Výborný , Y. Yahagi , J. Sinova , T. Jungwirth , J. Železný , Phys. Rev. Lett. 2021, 126, 127701. 33834809 10.1103/PhysRevLett.126.127701

[64] L. Šmejkal , J. Sinova , T. Jungwirth , (Preprint) arXiv:2105.05820, v2, submitted: Oct 2021.

[65] L. Šmejkal , A. B. Hellenes , R. González‐Hernández , J. Sinova , T. Jungwirth , Phys. Rev. X 2022, 12, 011028.

[66] L. Šmejkal , J. Sinova , T. Jungwirth , Phys. Rev. X 2022, 12, 040501.

[67] R. D. Zysler , D. Fiorani , A. M. Testa , L. Suber , E. Agostinelli , M. Godinho , Phys. Rev. B 2003, 68, 212408.

[68] H. M. Lu , X. K. Meng , J. Phys. Chem. C 2010, 114, 21291.

[69] G. P. Zhang , W. Hübner , G. Lefkidis , Y. Bai , T. F. George , Nat. Phys. 2009, 5, 499.

[70] A. M. Kalashnikova , A. V. Kimel , R. V. Pisarev , Phys.‐Usp. 2015, 58, 969.

[71] I. Sänger , V. V. Pavlov , M. Bayer , M. Fiebig , Phys. Rev. B 2006, 74, 144401.

