# Supplementary material for: Terahertz Spin Current Dynamics in Antiferromagnetic Hematite
Source: Adv Sci (Weinh). 2023 Apr 21;10(18):2300512. doi: 10.1002/advs.202300512 (PMC10288251; doi:10.1002/advs.202300512)
Supplement: Supplementary file 1 — Supporting Information [file ADVS-10-2300512-s001.pdf]

Supporting Information

Terahertz Spin Current Dynamics in Antiferromagnetic Hematite

Hongsong Qiu, Tom S. Seifert, Lin Huang, Yongjian Zhou, Zdeněk Kašpar, Caihong Zhang, Jingbo Wu, Kebin Fan, Qi Zhang, Di Wu, Tobias Kampfrath, Cheng Song,* Biaobing Jin,* Jian Chen, Peiheng Wu*

Section S1: Sample Characterization

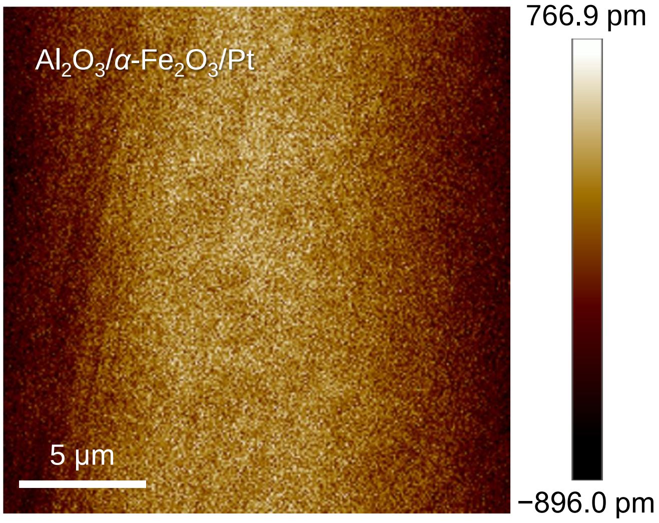

Figure S1. Smoothness of the sample surface. The smoothness of the α-Fe_2O_3/Pt surface is confirmed by atomic force microscopy (AFM). The film exhibits an atomically flat surface with a small roughness of ~0.1 nm.

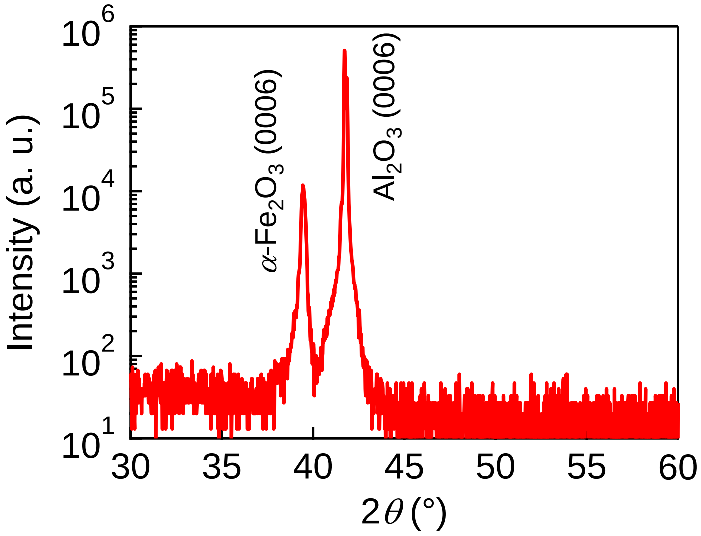

Figure S2. X-ray diffraction of the α-Fe_2O_3 film. X-ray diffraction (XRD) of the α-Fe_2O_3 film. There is a strong intensity peak corresponding to the (0006) plane, indicating the epitaxial growth of the α-Fe_2O_3 film on the Al_2O_3 substrate.

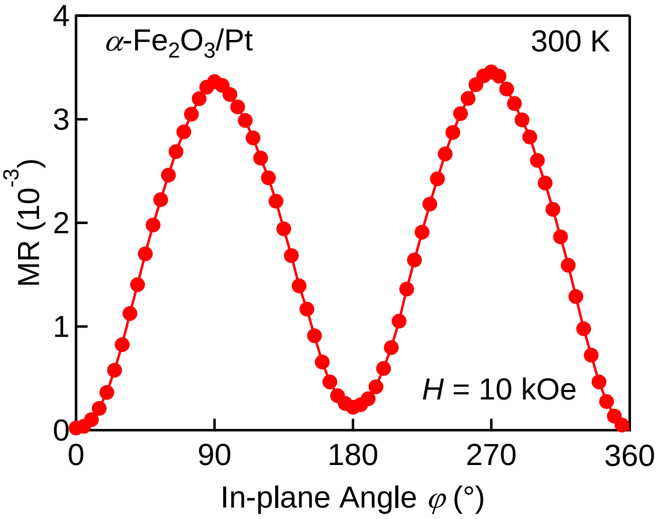

Figure S3. Spin Hall magnetoresistance. The longitudinal resistance R_{xx} of the α-Fe_2O_3/Pt sample at 300 K with a magnetic field $\boldsymbol{B}$ of 10 kOe is measured with the in-plane spin Hall magnetoresistance (SMR) scheme. The angle between the magnetic field $\boldsymbol{B}$ and the applied current $\boldsymbol{J}_c$ is denoted as φ. The phase shift of $\pi/2$ on the R_{xx}-curve for the α-Fe_2O_3/Pt sample along the φ-axis verifies the antiferromagnetic properties of the α-Fe_2O_3 film.

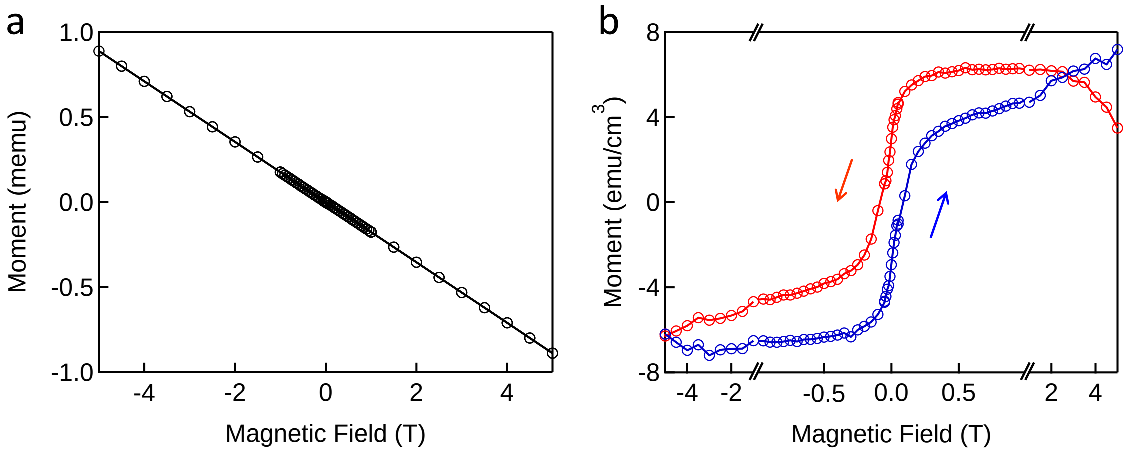

Figure S4. Hysteresis loops of the α-Fe_2O_3 film measured with a superconducting quantum interface device (SQUID) at 300 K. a) Raw SQUID data with a linear diamagnetic background from the Al_2O_3 substrate. b) The linear background in (a) is subtracted. The spin-flop transition happens at a magnetic field lower than 0.4 T. The magnetization $\boldsymbol{M}_s$ is around 6 emu/cm^3 near the spin-flop transition. Our measurements are highly consistent with previously reported values[1,2].

Section S2: Broadband THz Spectrum

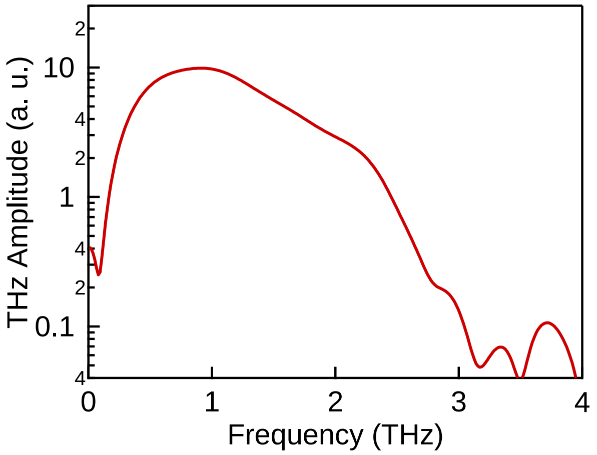

Figure S5. Broadband THz Spectrum. The THz spectrum for the amplified laser pump pulses covers the range from 0 to 3 THz, which is in contrast to the coherent behavior of the α-Fe_2O_3 magnon at ~ 0.2 THz[3,4].

Section S3: Dependence on the Pump Fluence

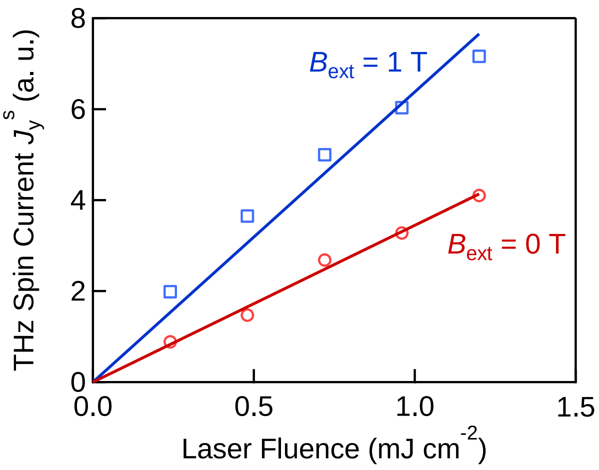

Figure S6. Dependence of the amplitude of J_y^s on the laser fluence for B_{ext} = 0 (red) and 1 T (blue). The experimental data (marks) are obtained by taking the peak values of the THz waveforms. The solid lines are linear fits to the experimental data. J_y^s is linearly proportional to the laser fluence and enhanced by a factor of ~2 by $\boldsymbol{B}_{ext}$ = 1 T. There is no significant saturation of J_y^s in the fluence range that we are concerned with. All the measurements are conducted with 100-fs laser pulses, wherein the two driving forces discussed in the main text have almost identical dynamics.

Section S4: β-dependence of J_y^s for α-Fe_2O_3/W

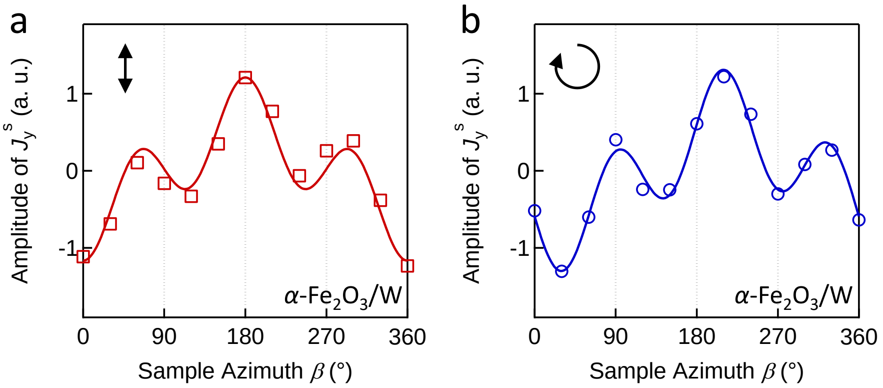

Figure S7. The amplitude of J_y^s for α-Fe_2O_3/W sample varies as a function of the sample azimuth β. There is a 30° shift for the curves obtained by using (a) the linear laser polarization and (b) the circular laser polarization. The marks are measured results and the solid curves are fit. The distortion of the curves is probably caused by the inhomogeneity of the α-Fe_2O_3 film.

Section S5: Hysteresis Loops for Various β

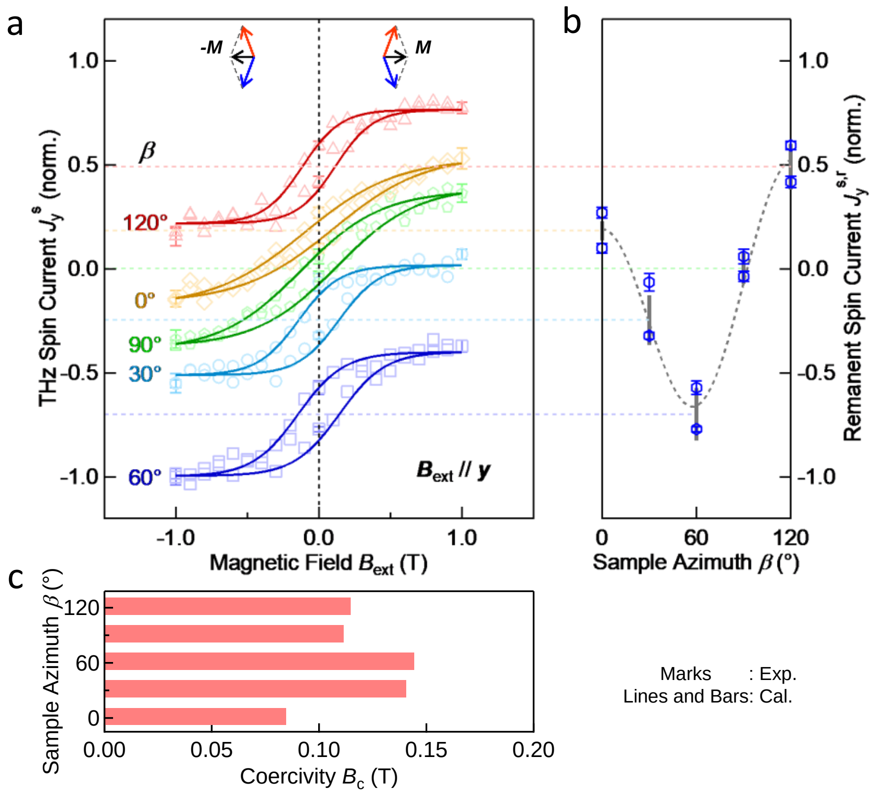

Figure S8. a) Hysteresis loops of J_y^s for various β at magnetic field $\boldsymbol{B}_{ext} \parallel \boldsymbol{y}$. The loop for β = 120° is the same as that in Figure 3a of the main text. b) The remanent THz spin current $J_y^{s,r}$ obtained in measurement (blue hexagon) and fit (grey bar). c) Coercive fields for various β extracted from the fitted hysteresis loop. Note that there is no artificial offset of the curves along the vertical axes.

As shown in Figure S8a, the evolution of the amplitude of the THz current with scanning $\boldsymbol{B}_{\text{ext}}$ forward and backward in the range from -1 T to 1 T when β is set at 0° (yellow diamond), 30° (cyan circle), 60° (blue square), 90° (green pentagon), and 120° (red triangle). The solid curves are a sigmoid fit for the hysteresis in the form of

$$J_y^{\text{s}}(B_{\text{ext}}) = \frac{C_{\text{asymp}}}{1+\mathrm{e}^{C_{\text{slp}}(-B_{\text{ext}}+C_{\text{coer}})}} + C_{\text{offset}}.$$

The parameter C_{asymp} determines the asymptote of the fit curve and is normalized to the maximum value. The parameter C_{slp} determines the slope of the fit curve. The parameter C_{coer} determines the coercivity. The as-measured offset of all the curves are fitted by the parameter C_{offset}. The dashed lines indicate the average values obtained during fitting. The remanent THz spin current $J_y^{\text{s,r}}$ is summarized in Figure S8b (measurement, blue hexagon; fit, grey bar). The dashed curve acts as a guide for the eye to demonstrate the evolution of $J_y^{\text{s,Aver}}$. As changing β. The period of 120° is consistent with the three-fold symmetry shown in Figure 2c of the main text. The coercive fields for various β can be extracted from the fitted hysteresis loop (Figure S8c). The coercivity lower than 0.15 T is estimated during the fitting of the hysteresis loop. The deviation between the coercivity for various sample azimuth β is probably caused by the slight as-grown inhomogeneity of our α-Fe_2O_3 film.

Section S6: Dependence of THz Spin Current on the Laser Polarization

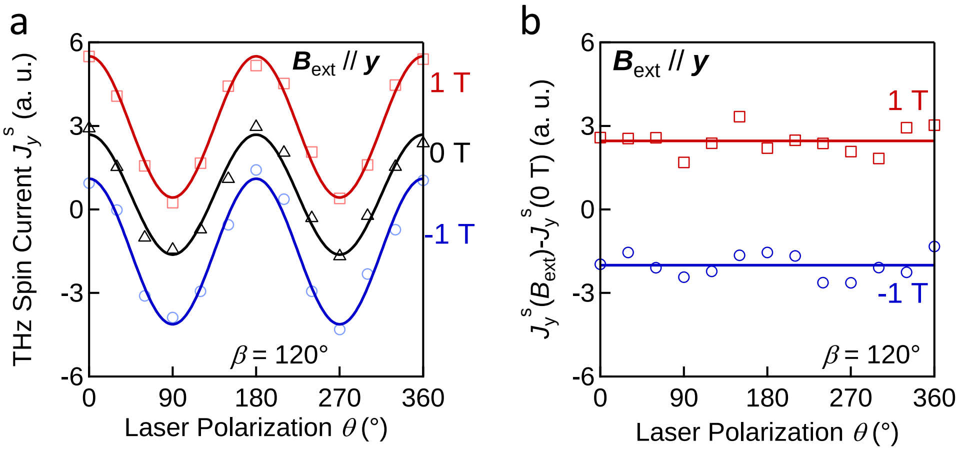

Figure S9. a) For $\boldsymbol{B}_{ext}$ = 0 T (black), the sign of J_y^s reverses as the laser polarization rotates from θ = 0° to 90°. When $\boldsymbol{B}_{ext}$ = 1 T (red) or -1 T (blue) is applied along $\boldsymbol{y}$, the dependences of J_y^s on θ move up or down compared to the case of $\boldsymbol{B}_{ext}$ = 0 T. b) The difference $J_y^s(B_{ext}) - J_y^s(0\text{ T})$ extracted from (a). The solid lines indicate the average of the experimental data. The definition of angle θ is given in Figure 1a of the main text. Note that the vertical location of all curves is determined by $|\boldsymbol{B}_{ext}| = 1$ T, but not artificially shifted for clarity.

Section S7: Reproducing the Odd Component of J_y^s with Typical Signals

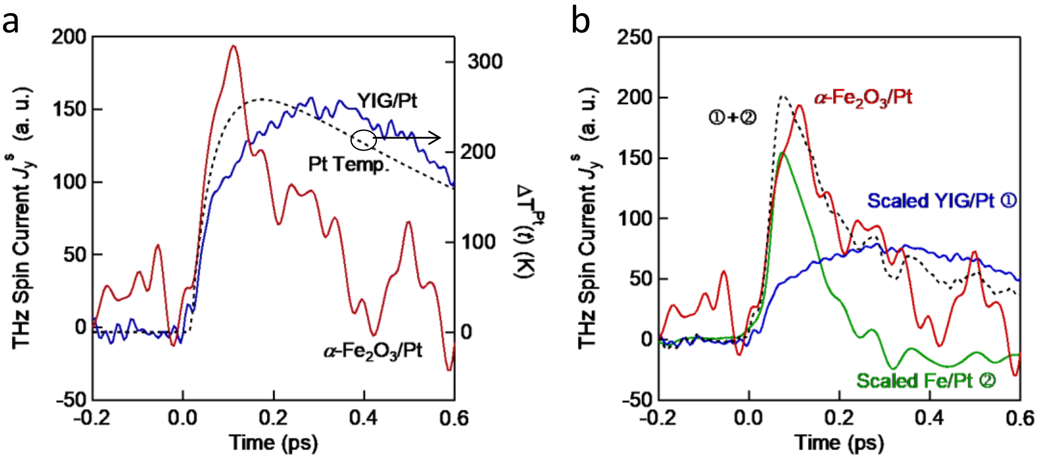

Figure S10. a) THz spin current J_y^s in α-Fe_2O_3/Pt (red) and YIG/Pt (blue). The evolution of the electron temperature in Pt is shown as a reference (dashed curve). The curves for J_y^s in α-Fe_2O_3/Pt and the temperature of Pt are the same as those in Figure 4b of the main text. The curves for J_y^s in YIG/Pt and the temperature of Pt are adapted from [5]. b) The temporal trace (dashed curve) that is obtained by linearly combining the scaled ultrafast spin currents in YIG/Pt (blue) and Fe/Pt (green) highly reproduces the odd component of J_y^s in α-Fe_2O_3/Pt (red).

Section S8: THz Spin Current for $\boldsymbol{B} \parallel \boldsymbol{x}$

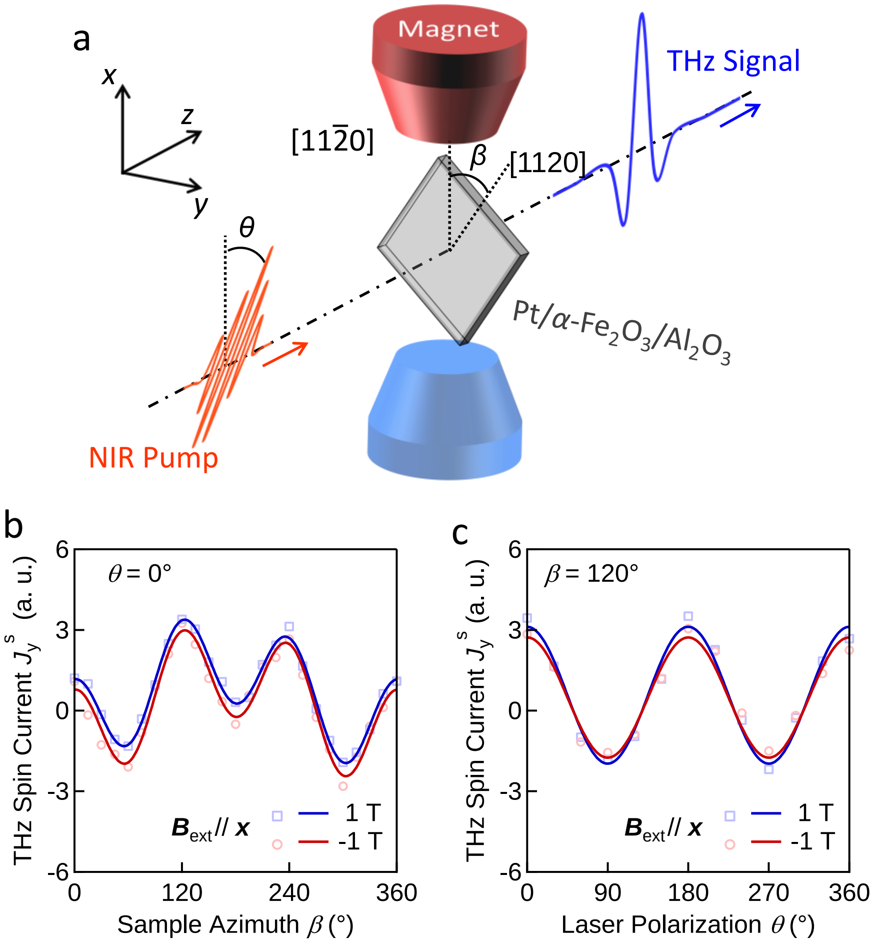

Figure S11. Impact of magnetic field $B_{\mathrm{ext}} \parallel x$ on J_y^{s}. a) Schematic of the transmission-type THz spectroscopy setup for $B_{\mathrm{ext}} \parallel x$. b) The relationship between the THz spin current J_y^{s} and the sample azimuth β for $B_{\mathrm{ext}} = \pm 1\mathrm{T}$. The laser polarization is fixed at $\theta = 0$. c) The impact of different magnetic fields on the evolution of J_y^{s} with the rotation of the laser polarization θ. The sample azimuth β is set at 120°. (Blue, 1 T; red, -1 T)

The ultrafast spin Seebeck effect does not contribute to $J_{\mathrm{y}}^{\mathrm{s}}$ when the magnetization $\boldsymbol{M}$ is parallel with the x axis[5], whereas the ISRS probably does because of the non-vanishing coefficient χ_{yyii}^{M} ($i = x$ or y). To confirm this, we set the magnetic field $\boldsymbol{B}_{\mathrm{ext}}$ along $\boldsymbol{x}$ and measure the dependence of $J_{\mathrm{y}}^{\mathrm{s}}$ on the sample azimuth β and laser polarization θ. The results shown in Figure S11b and S11c support the hypothesis: the three-fold (two-fold) symmetry in the relationship between $J_{\mathrm{y}}^{\mathrm{s,ISRS}}$ and β (θ) maintains under the magnetic fields $\boldsymbol{B}_{\mathrm{ext}} \parallel \boldsymbol{x}$, while there is barely vertical shift caused by the spin current $J_{\mathrm{y}}^{\mathrm{s,\Delta T}}$. There is a negligible deviation between the waveforms for B_{ext}=1 or -1 T both in Figure S11b and S11c. It is probably caused by the minor contribution of the deviation of the opto-magnetic coefficients for the ISRS process in the magnetic field.

Section S9: J_y^s of NiO/Pt and Py/Pt Structures

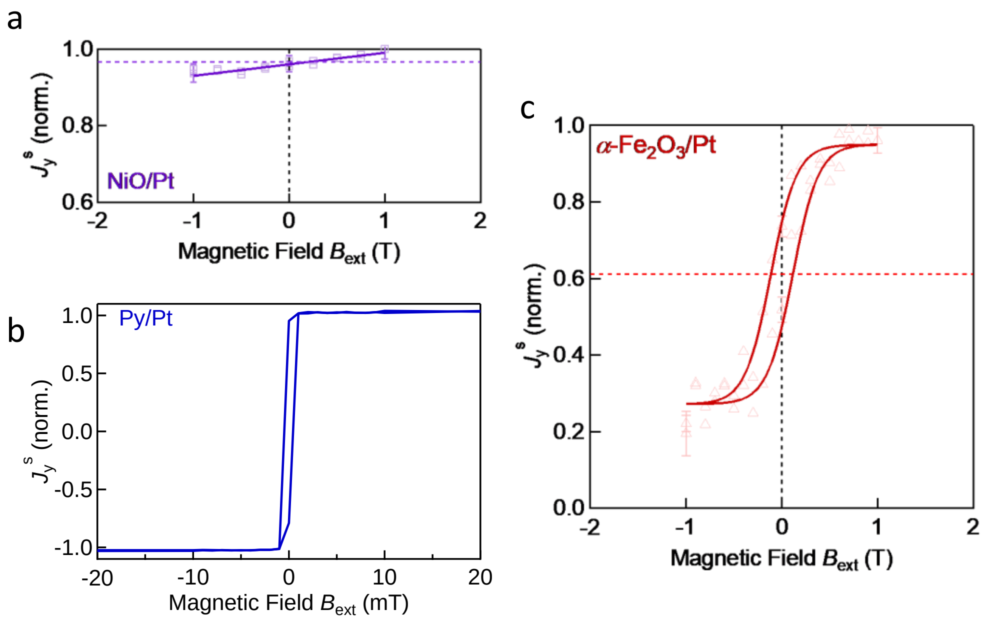

Figure S12. Dependence of the THz amplitude on the magnetic field for a) NiO/Pt, b) Py/Pt, and c) α-Fe_2O_3/Pt samples. The vertical axes are normalized to the maximum values.

The amplitude of the THz signal from the NiO/Pt sample is slightly influenced (< 7%) by the magnetic field up to 1 T (Figure S12a). The insensitivity of the NiO/Pt signal to the magnetic field is consistent with the large spin flop field of NiO[6]. A hysteresis loop can be observed when studying the dependence of the THz signal on the magnetic field for the Py/Pt sample as shown in Figure S12b. The coercive field of the Py (3 nm) is estimated to be less than 1 mT within the accuracy of our magnetic scanning. Both the performances of the NiO/Pt and Py/Pt samples are in stark contrast to that of the α-Fe_2O_3/Pt samples (Figure S12c).

Section S10. Theory of Magnetic Dynamics of α-Fe_2O_3

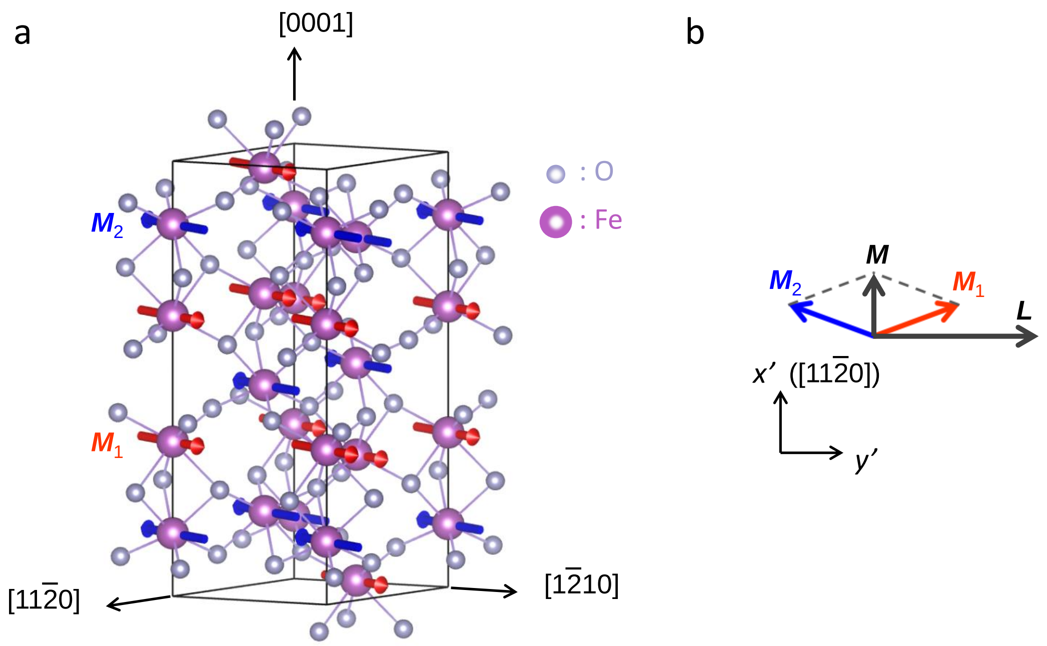

Figure ~~S12~~ S13. Crystallographic and magnetic structures of α-Fe_2O_3 at room temperature. a) The purple and grey spheres denote the Fe and O atoms, respectively. $\boldsymbol{M}_1$ (red arrows) and $\boldsymbol{M}_2$ (blue arrows) are the two spin sublattices. b) The two spin sublattices in α-Fe_2O_3 tilt towards the $\langle 11\bar{2}0\rangle$ axes at room temperature and the canting angle is exaggeratively illustrated. $(\boldsymbol{x}'\boldsymbol{y}'\boldsymbol{z}')$: the coordinate of the spin frame.

In this section, we focus on deducing the formula of the laser-induced effective magnetic fields $\boldsymbol{H}_{\mathrm{eff}}^{L}$ and $\boldsymbol{H}_{\mathrm{eff}}^{M}$. The symmetry analysis of hematite and the omission of some small physical quantities are used to simplify the derivation of the formula.

The hematite has a corundum-type crystal structure with a rhombohedral unit cell described by the point group $\bar{3}$m. The symmetry elements are 1, $\bar{1}$, $3(2_\perp)$, $3(\bar{2}_\perp)$, $\pm 3_z$, and $\pm\bar{3}_z$. At room temperature, the spins in each single spin domain of α-Fe_2O_3, for example, lie in the basal plane, pointing nearly parallel to $[1\bar{1}00]$ and $[\bar{1}100]$ axes. With this distribution of the spins, the direction [0001] is no longer the three-fold axes and the directions $[1\bar{2}10]$ and $[\bar{2}110]$ are no longer the two-fold axes. However, the direction $[11\bar{2}0]$ is still a two-fold axis. Therefore there are only symmetry elements 1, $\bar{1}$, $2_\perp$, and $\bar{2}_\perp$ left, such that the magnetic point symmetry is **2/m**[7].

After neglecting the terms of higher orders, ε_{ij} can be written as a power series in $\boldsymbol{M}$ and $\boldsymbol{L}$ with linear and quadratic coefficients

$$\varepsilon_{ij} = \varepsilon_{ij}^{0} + (k_{ijk}^{\mathrm{Re}} + ik_{ijk}^{\mathrm{Im}})M_k + (g_{ijkl}^{\mathrm{Re}} + ig_{ijkl}^{\mathrm{Im}})L_k L_l. \quad \text{(S1)}$$

The absence of absorption and the Onsager principle lead to the relation

$$\varepsilon_{ij}(\boldsymbol{M},\boldsymbol{L}) = \varepsilon_{ji}^{*}(\boldsymbol{M},\boldsymbol{L}) = \varepsilon_{ij}(-\boldsymbol{M},-\boldsymbol{L}). \quad \text{(S2)}$$

As a result, the real part k_{ijk}^{Re} and the imaginary part g_{ijkl}^{Im} vanish and the other coefficients satisfy $k_{ijk}^{\mathrm{Im}} = -k_{jik}^{\mathrm{Im}}$ and $g_{ijkl}^{\mathrm{Re}} = g_{jikl}^{\mathrm{Re}}$. For simplicity, we omit the superscripts Re and Im hereinafter. Besides, we focus on the spin domain whose local coordinate ($\boldsymbol{x'y'z'}$) coincides with that of the lab frame so that the prime is also omitted in the following contents for clarity. Some components in the coefficient tensor are zero in compliance with the magnetic symmetry. Furthermore, the tensor can be further simplified by taking into account the facts that L_y is always much larger than L_x and L_z and there is no laser component along z axis. Based on the above considerations, only k_{xyz} and $k_{yxz} = -k_{xyz}$ are the concerned third-rank coefficients. Similarly, the forth-rank coefficients are g_{yyyy}, g_{yyyz}, g_{yyzy}, g_{xxyy}, g_{xyxy}, g_{xyyx}, g_{xxyz}, g_{xxzy}, and the permutation of the Cartesian indices within the first two subscripts. According to Equation S2, all the fourth-rank coefficients obtained by permuting the first pair of subscripts and the second pair of subscripts are the same and we can subsume them into a single coefficient. Equation S1 is then rewritten as

$$\varepsilon_{ij} = \varepsilon_{ij}^{0} + ik_{ijk}M_k + g_{ijkl}L_kL_l. \tag{S3}$$

The effective magnetic fields induced by the ultrafast laser pulse via the nondissipative Raman-type mechanism can be written as the partial derivative of the Hamiltonian[8]:

$$\boldsymbol{H}_{\mathrm{eff}}^{L} = -\frac{\partial\mathcal{H}}{\partial\boldsymbol{L}} \text{ and } \boldsymbol{H}_{\mathrm{eff}}^{M} = -\frac{\partial\mathcal{H}}{\partial\boldsymbol{M}}. \tag{S4}$$

By applying Equation S3 into S4, the effective magnetic fields are expressed as

$$\boldsymbol{H}_{\mathrm{eff}}^{M} = \begin{bmatrix} 0 \\ 0 \\ -ik_{xyz}(E_xE_y^* - E_x^*E_y) \end{bmatrix} \tag{S5}$$

$$\boldsymbol{H}_{\mathrm{eff}}^{L} = \begin{bmatrix} -g_{xyxy}L_yE_xE_y^* - g_{yxyx}L_yE_x^*E_y \\ -g_{xxyy}L_yE_xE_x^* - g_{yyyy}L_yE_yE_y^* \\ -g_{xxyz}L_yE_xE_x^* - g_{yyyz}L_yE_yE_y^* \end{bmatrix} \tag{S6}$$

The torque imposed by the effective magnetic fields $\boldsymbol{H}_{\mathrm{eff}}$ causes an impulsive magnetization dynamics $\Delta\boldsymbol{M}(t)$, giving rise to the spin pumping on a time scale comparable to the ultrafast laser pulse.

Section S11. Fitting Parameters

In principle, the contribution of all α-Fe_2O_3 spin domains to the spin current can be calculated by transforming the tensors in each spin frame into that of the laboratory frame. The final form of the formula can be written in a phenomenological form $J_y^{\mathrm{s,ISRS}} \propto \mathrm{A}\cos(3\beta + B) + \mathrm{C}\cos(\beta)$. The fitting parameters determined for Figure 2c and 2d in the main text is shown below.

Table S1. Fitting Parameters.

Laser Polarization State	A	B	C
Vertical ($\theta = 0°$)	3.7	0°	-1.6
Horizontal ($\theta = 90°$)	-4.0	0°	-1.1
Right Circular	-3.1	90°	-2.0
Left Circular	3.2	90°	-1.4

References

[1] Y. Cheng, S. Yu, M. Zhu, J. Hwang, F. Yang, *Phys. Rev. Lett.* **2020**, *124*, 27202.

[2] P. Zhang, C. T. Chou, H. Yun, B. C. McGoldrick, J. T. Hou, K. A. Mkhoyan, L. Liu, *Phys. Rev. Lett.* **2022**, *129*, 17203.

[3] P. Stremoukhov, A. Safin, M. Logunov, S. Nikitov, A. Kirilyuk, *J. Appl. Phys.* **2019**, *125*, 223903.

[4] K. Grishunin, E. A. Mashkovich, A. V. Kimel, A. M. Balbashov, A. K. Zvezdin, *Phys. Rev. B* **2021**, *104*, 024419.

[5] T. S. Seifert, S. Jaiswal, J. Barker, S. T. Weber, I. Razdolski, J. Cramer, O. Gueckstock, S. F. Maehrlein, L. Nadvornik, S. Watanabe, C. Ciccarelli, A. Melnikov, G. Jakob, M. Münzenberg, S. T. B. Goennenwein, G. Woltersdorf, B. Rethfeld, P. W. Brouwer, M. Wolf, M. Kläui, T. Kampfrath, *Nat. Commun.* **2018**, *9*, 2899.

[6] F. L. A. Machado, P. R. T. Ribeiro, J. Holanda, R. L. Rodríguez-Suárez, A. Azevedo, S. M. Rezende, *Phys. Rev. B* **2017**, *95*, 104418.

[7] L. Oravova, Z. Zhang, N. Church, R. J. Harrison, C. J. Howard, M. A. Carpenter, *J. Phys. Condens. Matter* **2013**, *25*, 259501.

[8] A. Kirilyuk, A. V. Kimel, T. Rasing, *Rev. Mod. Phys.* **2010**, *82*, 2731.
